# Effects of emodin on inflammatory bowel disease-related osteoporosis

**DOI:** 10.1042/BSR20192317

**Published:** 2020-01-29

**Authors:** Jing-sheng Luo, Xinhua Zhao, Yu Yang

**Affiliations:** 1 Department of Orthopedics, First Affiliated Hospital of Gannan Medical University, Ganzhou 341000, Jiangxi Province, China; 2 Department of Gastroenterology, Mianyang Central Hospital, Mianyang 621000, SiChuan Province, China; 3 Department of Geriatrics, Affiliated Hospital of Guangdong Medical University, Zhanjiang 524001, Guangdong Province, China

**Keywords:** bone, emodin, Inflammatory bowel diseases, osteoclast, osteoporosis

## Abstract

Inflammatory bowel diseases (IBD) are related to bone loss. Emodin can influence the activity and differentiation of osteoblasts and osteoclasts. However, few studies have shown the effects of emodin on IBD-induced bone damage. The aim of the present study was to investigate the role of emodin in IBD-induced osteoporosis in an animal model. An IBD model in Sprague Dawley male rats was established by administering 2.5% dextran sulfate sodium (DSS) in the drinking water. Emodin was administered orally (30 mg/kg body weight) every other day starting in the third week for 9 weeks. Blood, colon and bone samples were obtained for biomarker assays and histological analysis. Bone biomechanical properties, microCT, metabolic biomarkers and bone histological changes were analyzed. The bone mass was significantly decreased, and the bone biomechanical properties and bone microstructure parameters of IBD rats were significantly worse than those of control rats (*P*<0.05). Tartrate resistant acid phosphatase staining also showed that the number of osteoclasts in bone in IBD rats were larger than that in bone in control rats. Emodin intervention abolished the changes in bone microstructure and biomechanical properties (*P*<0.05) induced by IBD. Osteoclast formation and serum C-terminal cross-linked peptide (CTX) and tumor necrosis factor α (TNF-α) were also inhibited by emodin (*P*<0.05). Emodin significantly abolished IBD-enhanced Traf6, NFATC1 and c-fos expression. Our data demonstrated that emodin suppresses IBD-induced osteoporosis by inhibiting osteoclast formation.

## Introduction

Inflammatory bowel diseases (IBDs) are a common cause of chronic gastrointestinal lesions. The incidence of IBD increased in the 1990s. Systemic inflammation and extraintestinal manifestations are usually observed in IBD patients [1,2]. The pathogenic mechanism of IBD is still not clarified. Genetic, immune and environmental factors may all play critical roles in IBD [3]. Secondary osteoporosis is an important extraintestinal manifestations [4]. Bone loss was observed in almost half of IBD patients [1]. In addition, the risk of bone fracture was relatively high in IBD patients. Systemic inflammation may be an etiological factor of bone loss in IBD patients. The cytokines released by inflammatory cells, like for example tumor necrosis factor α (TNF-α), can lead to mature osteoclast formation which causes excessive bone resorption and low bone mass [3].

There is a great need to prevent and treat IBD-related bone loss. Hormone replacement therapy, vitamin D supplementation, targeted therapies, as well as the direct treatment of inflammatory bone loss have been reported [5]. However, current therapies lead to remission in only a relatively small proportion of patients [5]. Therefore, it is vital to develop novel therapies [5]. Compounds derived from Asian herbs can protect against bone loss by promoting bone formation, inhibiting osteoclast formation or inducing osteoclast apoptosis [6–8]. Our previous study also indicated that natural products show great potential in protecting against IBD-induced bone loss [9]. Inflammation may be the main determinant of IBD-related bone loss [10]. Natural compounds with anti-inflammatory effects may be promising alternative agents for the treatment of IBD-related bone loss. Emodin, a type of anthraquinone compound, derived from the roots and bark of numerous plants of the genus Rhamnus, has potential pharmacological effects, such as anti-cancer and anti-inflammatory activities [7,11]. A few studies have also indicated that emodin may have potential for the treatment of osteoporosis by inhibiting bone resorption or promoting bone formation [12,13]. A recent study indicated that emodin can attenuate titanium particle-induced osteolysis by inhibiting osteoclastogenesis [14,15]. Excessive bone resorption is the critical reason for IBD-related bone loss. However, the role of emodin in IBD-induced bone loss remains unclear. We hypothesized that emodin may prevent IBD-induced bone loss by inhibiting osteoclastogenesis. In the present study, we showed the therapeutic effectiveness of emodin for IBD-induced bone loss in an IBD model.

## Materials and methods

### Experimental design

The *in vivo* studies were approved by the Institutional Animal Care Committee of First Affiliated Hospital of Gannan Medical University. The IBD models were induced by the administration of dextran sulfate sodium (DSS, Shanghai, China). Twenty-four 8-week-old Sprague Dawley male rats were housed in animal facilities under standard conditions (21 ± 1°C and a 12-h light–dark cycle).

We established the chronic IBD model using the methods described in a previous study [9]. Briefly, the rats received 2.5% DSS via their drinking water for 5 days, and then had free access to normal drinking water for 1 week. Subsequently, the rats were exposed to DSS for 5 days once again. Then, the animals had free access to normal water for 9 weeks. Rats in the control group were given normal drinking water for 12 weeks. Rats received emodin by gavage administration (30 mg/kg, Sigma, MO, U.S.A.) starting in the third week (three times per week). At the 12th week, the rats were killed for tissue collection. Blood was collected for the biomarker assay. Left femur and tibia were obtained for biomechanical assay and histological observation, respectively. The serum was separated from the blood and stored at −80°C. Bone tissue was stored at 4°C for less than 24 h prior to analysis.

### MicroCT analysis

The right tibia was collected for microCT analysis (Ge eXplore, GE healthcare, U.S.A.). The scan parameters were as follows [14]: tube voltages of 80 kV, current of 450 µA, field of view 2.5 cm and spatial resolution 45 × 45 × 45 μm. The region of interest (ROI) was set at 1.0 mm below the growth plate and extending for a longitudinal distance of 1 mm in the distal direction. The ROI was manually drawn and cortical bone was excluded from the analysis. A threshold of 800 was used during bone analysis by using Microview 2.5.0 software. The following data were obtained: bone volume to tissue volume ratio (BV/TV), trabecular thickness (Tb.Th), trabecular number (Tb.N) and trabecular separation (Tb.Sp).

### Biomechanical test

The biomechanical properties of the femur were determined using universal material testing machine (Instron 3300, MA). We performed a three-point bending test. Briefly, the soft tissues in the femur were removed, and then the femur was placed on the test platform. The test speed was set to 1.8 mm/min. The same operator performed all of the tests. We analyzed the following parameters: bending load, fracture strength and stiffness.

### Histological observations

We observed osteoclast formation by using histochemical staining. The protocol had been listed in the previous study [14]. Briefly, the soft tissue was removed and the tibia were fixed with 4% polyoxymethylene at 4°C for 48 h, followed by decalcification in 10% EDTA at 4°C for 1–2 weeks, paraffin embedding and sectioning. The sections used for osteoclast evaluation were dewaxed in dimethylbenzene three times (15 min each time), and then hydrated through a series of 100 to 40% ethanol solutions. Tartrate-resistant acid phosphatase (TRAP) staining was performed using a commercial kit following the manufacturer’s instructions (Sigma 387-A, St. Louis, U.S.A.). TRAP positive length and total TRAP positive area in trabecular bone were obtained using imaging software (SimplePCI, Compix Inc., Arizona, U.S.A.). We also measured the length and the area of trabecular bone. The relative TRAP positive length and area were calculated using the following equations: TRAP positive length/length of trabecular bone; TRAP positive area/area of trabecular bone. The sections of tibia were used for HE staining.

### Biomarker determination

Serum TNF-α and serum C-terminal cross-linked peptide (CTX) (IDS, U.K.) were determined by using enzyme-linked immunosorbent assay kits according to the manufacturer’s instructions. The intraassay and interassay differences were both lower than 6% for TNF-α and CTX.

### Real-time polymerase chain reaction

Total RNA was extracted from marrow-free tibias using TRIzol reagent. Real-time PCR was performed by using a commercial kit (SYBR PremixEx Tag kit, Takara, Japan). The relevant sense and anti-sense primers were listed as follows: Traf6: forward 5′agcccacgaaagccagaagaa’3, reverse 5′cccttatggatttgatgatga’3; NFATc1: forward 5′-CAACGCCCTGACCACCGATAG-3′, reverse 5′-GGCTGCCTTCCGTCTCATAGT-3′; c-fos: forward CGGGTTTCAACGCCGACTAC, reverse AAAGTTGGCACTAGAGACGGACAGA; GAPDH: forward 5′-ACCACAGTCCATGCCATCAC-3′, reverse 5′-TCCACCACCCTGTTGCTGTA-3′). The real-time polymerase chain reaction (RT-PCR) was perfomed at 94°C for 5 min and then for 35 cycles at 94°C for 5 s and 60°C for 20 s.

### Statistical analysis

Data are shown as the mean ± SD. One way analysis of variance (ANOVA) followed by a Bonferroni test were used for statistical analysis. *P*<0.05 was considered statistically significant.

## Results

### Bone microstructure

**Figure 1:**
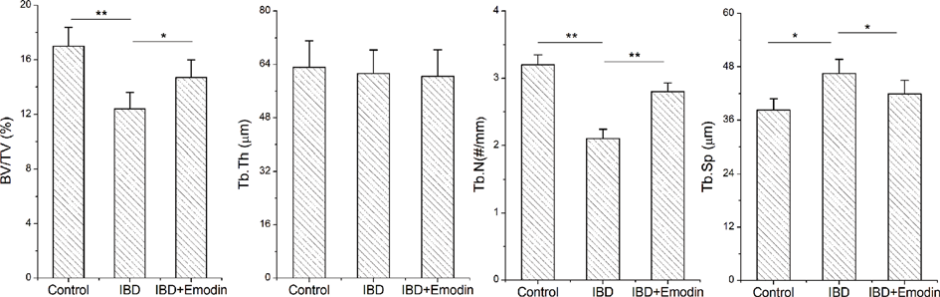
Bone microstructure Quantitative analysis of bone microstructure parameters in control and IBD rats with or without emodin, including bone volume fraction (BV/TV), Tb.N, Tb.Th and Tb.Sp. Values were showed as mean ± SD (*n*=8). **, *P*<0.01; *, *P*<0.05.

**Figure 2:**
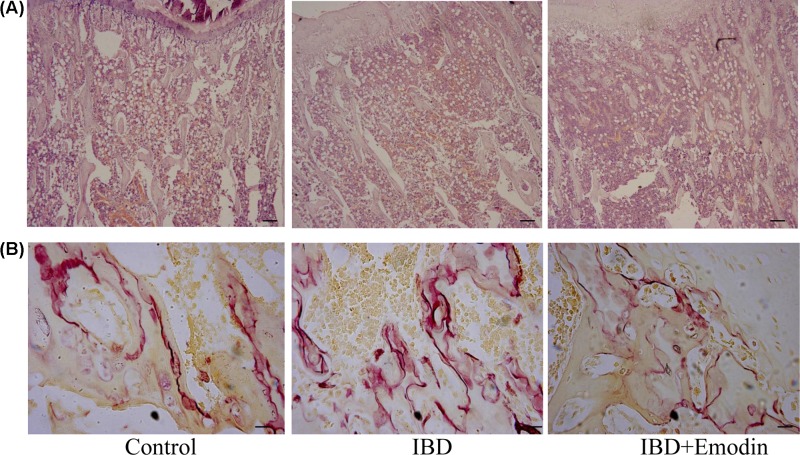
Histologic examinations Histologic feature of tibia using Hemotoxylin and Eosin staining (**A**) and TRAP staining (**B**). Emodin treatment protected the mice against bone loss induced by IBD as shown by increased Tb.N (A). The TRAP positive area was significantly decreased in IBD rats treated with emodin compared with those without emodin intervention (B). Scale bar 100 μm.

The data of bone microstructure and histologic examinations are displayed in Figures 1 and 2A, respectively. Qualitative analysis of HE staining, and quantitative data from MicroCT analysis both showed bone loss in IBD rats compared with control rats, and decrease in Tb.N and the number of conjunction points, and an increase in Tb.Sp were observed. Emodin treatment inhibited the bone loss induced by IBD. The BV/TV and Tb.N were increased and Tb.Sp was deceased in emodin-treated rats compared with the values in IBD rats (*P*<0.05 or 0.01).

### TRAP positive cells in bone tissues

**Figure 3:**
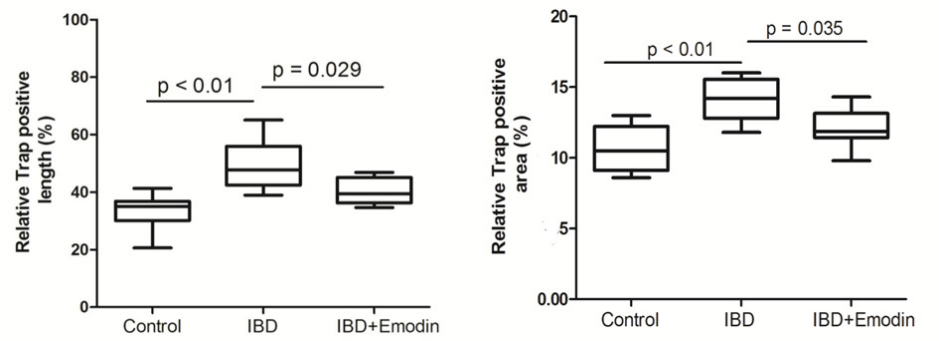
Effects of emodin on osteoclast formation (*n*=8) The TRAP positive area and length in emodin-treated rats was larger than the control. Emodin suppressed osteoclast formation in the tibia of IBD rats.

Osteoclast formation was evaluated by the TRAP positive tissues. The osteoclast formation in IBD rats was markedly higher than that in the control (Figures 2B and 3). The relative TRAP positive area and length were higher in IBD rats than that in control rats (*P*<0.01). The TRAP positive area and length in emodin group were smaller than those in IBD group (*P*<0.05).

### Biomechanical properties

**Figure 4:**
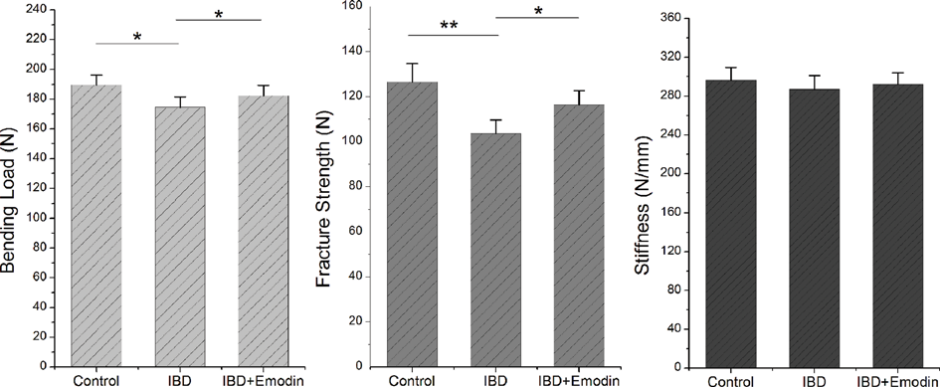
Effects of emodin on bone biomechanical properties (*n*=8) The following parameters were tested using a universal materials test machine: bending load, yield strength and stiffness. The bending load and fracture strength in IBD rats receiving emodin were both increased compared with those without emodin intervention. **, *P*<0.01; *, *P*<0.05.

The mechanical properties of the femur are shown in Figure 4. Bone of IBD rats showed lower bending load, fracture strength and stiffness than those in the control (*P*<0.05). The bending load and fracture strength in emodin group were significantly higher than those in the IBD group (*P*<0.05), but no significant difference was observed in stiffness.

### Biomarkers

**Figure 5:**
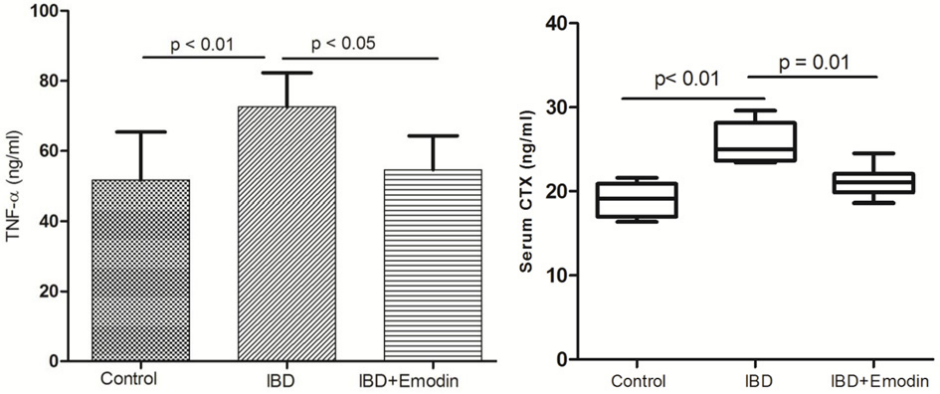
Serum TNF-α and CTX levels in control, IBD and emodin-treated IBD rats (*n*=8) The TNF-α and CTX levels were significantly higher in IBD rats than those in control. The TNF-α and CTX level was significantly decreased in IBD rats treated with emodin.

The serum TNF-α was significantly higher in DSS-treated rats (IBD group) than that in control rats (*P*<0.01). Serum CTX is a biomarker reflecting osteoclast-related bone resorption. The serum CTX level of IBD rats was obviously higher than that in control rats (*P*<0.05) (Figure 5). The levels of CTX and TNF-α in rats receiving emodin were significantly lower than those in IBD rats (*P*<0.05).

### mRNA expression

**Figure 6:**
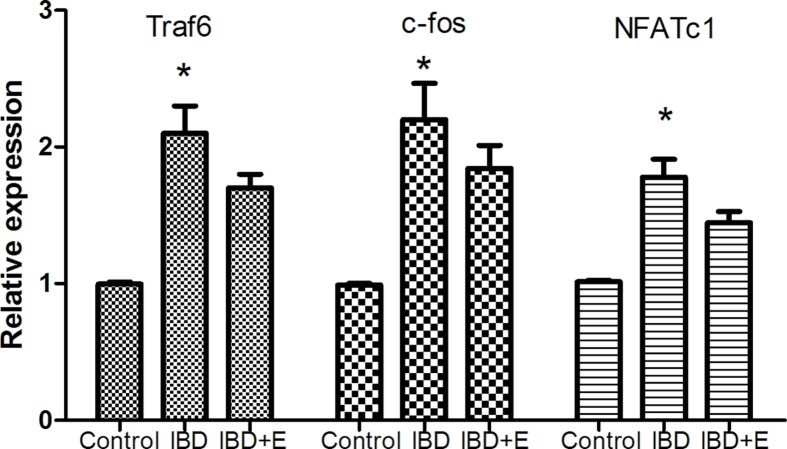
Emodin inhibited mRNA expressions of TRAF6, NFATc-1 and c-fos in IBD rats (*n*=8) *, *P*<0.05 or 0.01 versus other groups.

The Traf6, c-fos and NFATC1 mRNA expression in bone of IBD rats was obviously higher than that in bone of control rats (*P*<0.05) (Figure 6). The mRNA expression in bone of emodin group was significantly lower than that in bone of IBD rats (*P*<0.05).

## Discussion

Bone loss and osteoporosis are common extraintestinal manifestations in IBD patients [16]. A total of 40–50% of IBD patients may have bone loss and IBD patients have high risk of bone fractures [4]. Study *in vivo* indicated that natural compounds derived from herbs are applicable for the treatment of IBD-related osteoporosis [9]. In the present study, our data showed that emodin inhibited IBD-induced bone loss by inhibiting osteoclastic bone resorbtion.

Inflammation may affect bone cells. The inflammatory response can activate an array of cytokines that can affect the differentiation and function of osteoclasts and osteoblasts [5]. Therefore, steroid or anti-TNF-α monoclonal antibodies are useful for the treatment of IBD-induced bone loss [17]. However, these drugs usually have significant adverse effects. It is vital to develop novel therapies [5]. Previous studies have shown that natural compounds derived from herbs are available for the treatment of osteoporosis by promoting bone formation or inhibiting bone resorption [6,8,12]. Emodin is a natural compound and has shown great potential in anti-inflammatory [7], anti-cancer [18], anti-hyperglycemic [19] and anti-oxidant activities [11]. Several studies also reported the therapeutic effect of emodin on bone loss. Emodin enhances bone formation by promoting osteoblast differentiation or mineralization [12,20]. Moreover, emodin can also inhibit osteoclast formation or activity induced by LPS exposure and titanium particles [15,21]. Interestingly, our data indicated that emodin could improve bone status in IBD rats, as shown by its improvement of bone microstructure parameters and bone biomechanical properties. Emodin may have therapeutic effects in the treatment of IBD-related osteoporosis.

Excessive osteoclast formation is one of the critical reasons for IBD-induced bone loss. Serum TNF-α was shown to be elevated in the presence of several extraintestinal manifestations [22]. TNF-α enhanced receptor activator of nuclear factor-κ B ligand (RANKL) expression or cooperated with RANKL to promote osteoclast formation [23]. RANKL/RANK pathway is critical for osteoclasts formation and differentiation [24]. Emodin intervention inhibited IBD-induced osteoclast formation. However, the possible underlying mechanisms remain unclear. Kim and colleagues [13] indicated that emodin inhibits the gene expression of osteoclast differentiation, such as c-fos and NFATc1. Our data also showed that Traf6, c-fos and NFATc1 mRNA expression was inhibited by emodin. In addition, previous study indicated that emodin affects IKKb activation and suppresses NF-ƙB transcriptional activation [15], which indicated that emodin may decrease osteoclast formation by inhibiting the RANKL-associated signal pathways. In addition, Hwang et al. [6] showed that emodin can suppress inflammatory response in collagen-induced arthritic mice. In addition, TNF-α may cooperate with RANKL to stimulate osteoclast formation [25]. Our data also showed that emodin inhibited IBD-induced increase in TNF-α. Therefore, emodin may inhibit IBD-related bone loss by its anti-inflammatory effects. Emodin may enhance the osteoblast differentiation and mineralization [12]. However, the inhibition on osteoclast formation may be the main mediating factor because excessive osteoclast formation is the critical reason for IBD-related bone loss.

Several limitations do exist in our study. First, we did not show the effects of emodin on IBD because the focus of the present study is the effects of emodin on IBD-related bone loss and previous studies have shown that emodin may ameliorate dextran sodium sulfate-induced colitis [26]. Second, we did not show the roles of emodin in normal bone and bone formation because excessive osteoclast formation is the critical reason for IBD-related bone loss. The role of bone formation could not be excluded because emodin can stimulate osteoblast formation [21]. Finally, the potential molecular mechanisms were not investigated in this *in vivo* study.

Our data indicated that emodin, a natural compound derived from herbs, may protect against IBD-induced bone loss by inhibiting osteoclast formation. Emodin may have therapeutic potential for the treatment of IBD-related bone loss.

## Abbreviations

BV/TV: bone volume to tissue volume ratio
CTX: C-terminal cross-linked peptide
DSS: dextran sulfate sodium
HE: hematoxylin-eosin
IBD: inflammatory bowel disease
LPS: lipopolysaccharide
microCT: micro-computed tomography
RANKL: receptor activator of nuclear factor-κ B ligand
ROI: region of interest
SD: standard deviation
Tb.Th: trabecular thickness
Tb.N: trabecular number
Tb.Sp: trabecular separation
TNF-α: tumor necrosis factor α
TRAP: tartrate-resistant acid phosphatase
